# Avian influenza and coronaviruses in live animal and wet markets in Laos: prevalence and public health considerations

**DOI:** 10.3389/fcimb.2026.1786183

**Published:** 2026-04-07

**Authors:** Patrick Höller, Elin Asp, Julia Pärssinen, Vannaphone Phouthana, Nittakone Soulinthone, Soukangna Keopaseuth, Kaisone Chanda, Jiaxin Ling, Johanna F. Lindahl, Mahmoud M. Naguib

**Affiliations:** 1 Zoonosis Science Centre, Department of Medical Biochemistry and Microbiology, Uppsala University, Uppsala, Sweden; 2 Faculty of Agriculture, National University of Laos, Vientiane, Lao People's Democratic Republic; 3 Department of Animal Health and Antibiotic Strategies, Swedish Veterinary Agency, Uppsala, Sweden; 4 Department of Infection Biology and Microbiomes, Institute of Infection, Veterinary and Ecological Sciences, University of Liverpool, Liverpool, United Kingdom

**Keywords:** avian influenza virus, coronavirus, Laos, live animal markets, one health

## Abstract

**Background:**

Live animal and wet markets (LWM) serve as critical interfaces where humans closely interact with domestic and peri-domestic animals, facilitating the spillover of zoonotic pathogens. Previous outbreaks of avian influenza viruses (AIV) and coronaviruses (CoV) linked to these markets underscore their significant public health risks. Despite the high density of LWM and historical viral spillovers in Southeast Asia, studies on the prevalence of respiratory viruses in LWM in Laos remain limited.

**Methods:**

To address this gap, we conducted a study across 20 LWM in the capital region of Laos in 2023. A total of 266 oropharyngeal swab samples from live and slaughtered ducks and chickens, the environment, and the air, were collected and screened for AIV and CoV. Furthermore, a questionnaire assessed the knowledge and attitudes of vendors and shoppers regarding disease risks at LWM.

**Results:**

We found a higher prevalence of AIV in swabs from live ducks (72%) compared to chickens (18%), while CoV was more common in chickens (13%) than ducks (5%). Air samples showed a prevalence of 38% for AIV and 8% for CoV. Subtyping of AIV revealed the circulation of the high pathogenicity H5N1 strain which is genetically characterized as clade 2.3.2.1e.

**Conclusions:**

These findings highlight the significant public health risks associated with LWM in Laos and emphasize the need for continuous surveillance and control measures to mitigate the risk of future zoonotic outbreaks.

## 1 Introduction

Live animal and wet markets (LWMs) are informal markets selling perishable food and live animals and play a crucial role in the food supply chain and the economy of many countries, particularly in Southeast Asia (SEA) (Naguib et al., 2021). Despite their benefits, LWMs pose several public health risks and have been associated with spillover events of several zoonotic diseases as exemplified by the COVID-19 pandemic (Lin et al., 2021). LWMs provide an optimal environment for close interactions, leading to high risk of cross-species transmission of pathogens between humans and domestic-, peri-domestic, as well as wild animals. At least 75% of all emerging human diseases are of zoonotic origin, e.g. previous outbreaks of coronaviruses (CoV) and avian influenza viruses (AIV), which have crossed the species barrier from animal hosts to humans. Hence, environments where humans and animals are in close proximity, such as LWMs, may facilitate opportunities for pathogen spillover and therefore warrant further investigation (Taylor et al., 2001; Ellwanger and Chies, 2021; Lin et al., 2021; Naguib et al., 2021).

The natural reservoirs of influenza A viruses are wild aquatic birds, however, the virus can infect other species including domesticated birds, pigs, horses, dairy cows, and is associated with sporadic infection to humans (CDC, 2023). Since the start of the 20^th^ century, there have been nine pandemics in total, four of which were caused by an influenza A virus (1918, 1957, 1968, 2009), which all had an avian or swine origin and spilled over to humans [6,7]. Globally, since 1997, there have been 896 cases of reported human infections with influenza A virus (A/H5N1) with 463 deaths, in addition to 92 human cases with H5N6, including 37 deaths (World Health Organization, 2024). The majority of those cases were located in East and Southeast Asia (Lai et al., 2016). Highly pathogenic avian influenza virus (HPAIV) was first reported in poultry in Laos in 2003-2004 (Boltz et al., 2006). Since 2005, there have been three reported cases of A/H5N1 in Laos, with at least two fatalities. The latest report is from 2020, where the infected persons had lived in close contact with domestic poultry (World Health Organization, 2020). Additionally, one human case with A/H5N6 was documented in Laos, and A/H5N6 has also been found in ducks, which the infected patient had been in contact with (Sengkeopraseuth et al., 2022). Another AIV, H7N9, has been detected in different bird species in SEA since its first record in 2013 (Millman et al., 2015).

As mentioned above, coronaviruses (CoVs) belong to the family *Coronaviridae* and are another group of viruses associated with live animal and wet markets (LWMs) and zoonotic spillover events. CoVs have a wide host range, including mammals, and avian species (V’kovski et al., 2021). The first coronavirus was reported from baby chicks in USA and designated as the avian coronavirus (infectious bronchitis virus (IBV)) (Schalk et al., 1931). This virus is known to cause highly transmissible upper respiratory disease in domestic chickens (*Gallus gallus domesticus*), which can negatively impact egg and meat production and thereby affecting the poultry industry (Sjaak de Wit et al., 2011). Despite the existence of a vaccine against IBV, it continues to spread and cause several outbreaks globally. One of the reasons for this is the low cross-protection between different strains (Al-Khalaifah et al., 2022). Furthermore, other viruses from the family Coronaviridae have caused major outbreaks in mammalian species than birds including: two epidemics, severe acute respiratory syndrome (SARS) caused by SARS-CoV-1, Middle East respiratory syndrome (MERS) caused by MERS-CoV, and one pandemic, COVID-19 caused by SARS-CoV-2, over the last two decades (European Centre for Disease Prevention and Control, 2022).

A robust understanding of infectious diseases is generally associated with an increased likelihood of engaging in preventive measures against such infections (Golden et al., 2021; Kundu et al., 2021). This is particularly important for individuals at greater risk of zoonotic transmission, such as livestock farmers or fresh meat market vendors (Ngoshe et al., 2022; Wallace et al., 2022). However, a previous study conducted in Laos revealed that vendors showed both low disease awareness as well as poor use of protective equipment (Philavong et al., 2020). The aim of this study was to investigate the prevalence of AIV and CoV in poultry and environmental samples collected from LWMs in the greater Vientiane province in Laos. Additionally, a survey questionnaire was conducted with shoppers and vendors at the same LWMs. The survey addressed the knowledge about the disease, preventive measures, and vaccination. The findings of this study provide a better understanding of zoonotic spillover risks of AIV/CoV in LWMs in Laos and SEA.

## 2 Materials and methods

### 2.1 Live wet markets and sample collection

**Figure 1:**
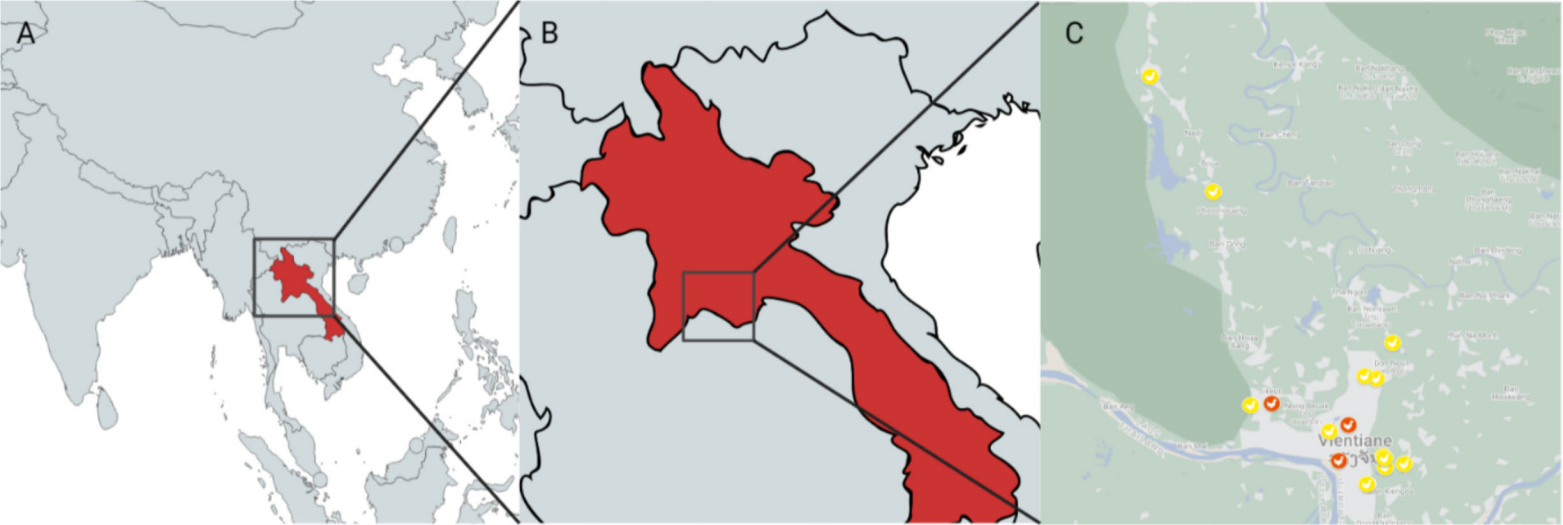
**(A)** shows the location of Laos in the region of Southeast Asia. **(B)** shows a close view of Laos and the location of the capital Vientiane, and the province Vientiane. In **(C)** the individual markets can be seen within the province and the capital Vientiane. Yellow icons show markets with only live poultry; red icons show markets with slaughtered poultry.

The sampling of this cross-sectional study was conducted during the first quarter of 2023. In total 20 markets were surveyed, four located in the province of Vientiane, and 16 in Vientiane Capital in Laos Peoples Democratic Republic. The location of Laos as well as the geographical distribution of the individual markets is illustrated in **Figure 1**. An ethical permit was obtained from the Lao national ethics committee (number 032/NECHR, dated January 6, 2023). All participants were approached individually, and the questionnaire was conducted after they gave verbal informed consent. During sampling, the team was always accompanied by provincial and district health staff or market directors. The sampling of poultry was only conducted after the vendors/owners were informed about the process and gave verbal informed consent. The aim was 270 samples, which would allow the estimation of a prevalence of 20% 5% precision and 95% level of confidence (a suggested sample size of 246, plus small design effect) (Naing et al., 2022).

The presence of live chickens and/or ducks and/or slaughtered chickens served as inclusion criteria for the markets. Markets were categorized according to the estimated total number of vendors into small markets (1 to 14 vendors), medium markets (15 to 100 vendors), and large markets (more than 100 vendors). Depending on the market type, one to five bird market stalls were selected for sampling. Market selection was guided by provincial officials as well as veterinary staff from the agricultural faculty of the National University of Laos (NUoL). The 20 markets were selected based on size, species diversity, and accessibility, in consultation with local authorities. Within the markets, vendors and buyers were selected from different locations within each market. To ensure that the sample represented the whole market, neighbours were avoided, and vendors were chosen from across all areas of the market. This was particularly important in larger markets.

Oropharyngeal swabs were taken from live and slaughtered poultry using a flocked cotton swab and preserved in viral transport media. Swabs were obtained gently by authorized personnel “trained veterinary staff and research team members certified for sample collection” without euthanasia. Swabs from slaughtered poultry were only taken if live animals were not available. From all markets, air samples were obtained using an air sampler with compatible sampling chambers (AeroCollect^®^; Indical Bioscience; Brøndby, Denmark) for a minimum of five minutes in close proximity to bird cages. Environmental swabs (swab of cage surface) were allowed to be taken only from three markets (**Table 1**). All samples, swabs, and air-sample chambers were kept in a cooling box until transfer to -80 °C or direct analysis.

**Table 1:** Detailed positive rates of AIV and CoV across various sample types for each individual market. n.a.: non-applicable, no samples of this type were collected at the corresponding market. .

Market	Market type	% Positive for CoV (# of positives/# of samples)					% Positive for AIV (# of positives/# of samples)				
		Air Sample	Environmental Swab	Slaughtered Poultry	Live Ducks	Live Chicken	Air Sample	Environmental Swab	Slaughtered Poultry	Live Ducks	Live Chicken
1	Medium	0% (0/5)	0% (0/3)	n.a.	0% (0/7)	0% (0/5)	20% (1/5)	67% (2/3)	n.a.	86% (6/7)	0% (0/5)
2	Small	0% (0/1)	0% (0/1)	n.a.	0% (0/2)	0% (0/3)	100% (1/1)	100% (1/1)	n.a.	100% (2/2)	0% (0/3)
3	Small	0% (0/1)	n.a.	n.a.	0% (0/1)	0% (0/3)	100% (1/1)	n.a.	n.a.	100% (1/1)	100% (3/3)
4	Medium	0% (0/1)	n.a.	n.a.	0% (0/2)	18% (2/11)	0% (0/1)	n.a.	n.a.	0% (0/2)	9% (1/11)
5	Small	0% (0/1)	0% (0/3)	n.a.	0% (0/4)	0% (0/6)	100% (1/1)	100% (3/3)	n.a.	50% (2/4)	17% (1/6)
6	Medium	0% (0/1)	n.a.	0% (0/9)	n.a.	n.a.	0% (0/1)	n.a.	0% (0/9)	n.a.	n.a.
7	Large	100% (1/1)	n.a.	0% (0/8)	n.a.	0% (0/2)	0% (0/1)	n.a.	0% (0/8)	n.a.	50% (1/2)
8	Small	0% (0/1)	n.a.	n.a.	0% (0/2)	0% (0/5)	100% (1/1)	n.a.	n.a.	100% (2/2)	0% (0/5)
9	Small	0% (0/1)	n.a.	n.a.	0% (0/2)	n.a.	0% (0/1)	n.a.	n.a.	50% (1/2)	n.a.
10	Medium	100% (1/1)	n.a.	n.a.	25% (1/4)	32% (6/19)	0% (0/1)	n.a.	n.a.	100% (4/4)	5% (1/19)
11	Large	0% (0/1)	n.a.	0% (0/6)	n.a.	n.a.	100% (1/1)	n.a.	17% (1/6)	n.a.	n.a.
12	Large	0% (0/1)	n.a.	0% (0/24)	n.a.	n.a.	0% (0/1)	n.a.	50% (12/24)	n.a.	n.a.
13	Small	0% (0/1)	n.a.	n.a.	22% (2/9)	80% (8/10)	0% (0/1)	n.a.	n.a.	78% (7/9)	0% (0/10)
14	Small	0% (0/1)	n.a.	n.a.	0% (0/6)	n.a.	100% (1/1)	n.a.	n.a.	67% (4/6)	n.a.
15	Small	0% (0/1)	n.a.	n.a.	0% (0/8)	3% (1/38)	100% (1/1)	n.a.	n.a.	75% (6/8)	11% (4/38)
16	Large	0% (0/1)	n.a.	n.a.	n.a.	n.a.	0% (0/1)	n.a.	n.a.	n.a.	n.a.
17	Large	0% (0/1)	n.a.	n.a.	n.a.	0% (0/5)	0% (0/1)	n.a.	n.a.	n.a.	0% (0/5)
18	Small	0% (0/1)	n.a.	n.a.	n.a.	0% (0/15)	0% (0/1)	n.a.	n.a.	n.a.	73% (11/15)
19	Small	0% (0/1)	n.a.	n.a.	0% (0/6)	n.a.	100% (1/1)	n.a.	n.a.	83% (5/6)	n.a.
20	Small	0% (0/1)	n.a.	n.a.	0% (0/4)	0% (0/5)	0% (0/1)	n.a.	n.a.	25% (1/4)	20% (1/5)
	Total	8% (2/24) 95% CI 1-27%	0% (0/7) 95% CI^#^ 0-41%	0% (0/47) 95% CI 0-8%	5% (3/57) 95% CI 1-15%	13% (17/127) 95% CI 8-21%	38% (9/24) 95% CI 19-59%	86% (6/7) 95% CI 42-99%	28% (13/47) 95% CI 16-43%	72% (41/57) 95% CI 58-83%	18% (23/127) 95% CI 11-26%

*Additionally, 2 of 4 live guinea fowls tested positive for AIV.

#CI is confidence intervals

Additionally, at 19 out of 20 markets, up to 11 vendors and/or customers were selected if they shopped at the selected shop or a nearby shop, and interviews using a structured questionnaire were conducted as provided in the **Supplementary Material**. None of the questions were mandatory, and all could be skipped individually if the respondents preferred to.

### 2.2 Molecular detection of avian influenza and corona virus

Air samples were eluted from the AeroCollect sample chambers by using a modified protocol, first described by Leding et al (Leding et al., 2022), where 30 µL nuclease-free water were added to the sample chamber, swirled around 20 times, and then the liquid was collected. The eluates of the sample chambers were directly used for PCR without the need for RNA extraction, as advised by the manufacturer. Further, the RNA of the swab-eluates was extracted using the QIAamp Viral RNA Mini Kit (Qiagen; Hilden, Germany) according to the manufacturer’s instructions with slight modifications. A total of 30 µL nuclease-free water were used to elute the RNA in the last step. The extracted RNA was stored at -80 °C until further analysis.

Three different RT-qPCRs were used to detect 1. AIV; 2. pan-CoV; 3. SARS-CoV-2 particularly. The SARS-CoV-2 protocol was only applied to air samples. The reverse transcription, quantitative PCR (RT-qPCR) assays were performed using AgPath-ID™ One-Step RT-PCR Kit (Thermo Fischer Scientific, Waltham, Massachusetts, USA), a qTower^3^ thermocycler. For the detection of AIV, a protocol from Spackman et al (Spackman, 2020). was used targeting the M gene segment. Briefly, a set of primers and probes, described at **Supplementary Table 1**, were used. Further, a TaqMan RT-PCR assay described previously Muradrasoli et al (Muradrasoli et al., 2009). was used for detection of CoV. Additionally, for the detection of SARS-CoV-2, a protocol from *Corman* et al (Corman et al., 2020). was applied for all collected air samples. Sequences for all nucleotide-based reagents are provided in **Supplementary Figure 1**. Ct values of Avian Influenza Virus (AIV) and Coronavirus (CoV) detected in different sample types. Each point represents an individual positive qPCR result for either AIV (triangles) or CoV (circles) across all sample types.

### 2.3 Subtyping and genetic characterization

An additional PCR was conducted on all positive AIV samples with a Ct-value below 30 using primers to amplify all eight influenza gene segments according to King et al (King et al., 2022). The extracted RNA and PCR product was then placed on QIAcard FTA Classic cards (Qiagen; Hilden, Germany) according to the manufacturer’s instructions and shipped to Uppsala University for further subtyping and sequencing. To elute the RNA/PCR product from the FTA cards, a 4 mm^2^ square was incubated in 100 µL TE buffer (10 mM Tris, 1 mM EDTA, pH 8) at 70 °C for 1 h with vortexing at 10 min intervals.

Any sample with a Ct value less than 40 is considered a “positive” detection. Subtyping of the haemagglutinin gene of AIV was performed on a total of 31 positive influenza M samples. Samples were selected based on the virus load and sample quality typically samples with Ct value being <30 in the M RT-PCR, ensuring the most representative dataset within the scope of our study. TaqMan RT-PCR specific assays for each A/H5, A/H7, and A/H9 were conducted. The RT-qPCRs were conducted according to Hassan et al (Hassan et al., 2022). using the CFX Connect Real-Time System, CFX96 Real-Time System, the CFX connect RT-PCR detection system (BioRad; Hercules, CA, USA), and using AgPath-ID™ One-Step RT-PCR Kit (Thermo Fischer Scientific, Waltham, MA, USA).

Further, ten samples were selected for whole-genome amplification based on virus load and sample quality (RNA concentration and it is degraded or not) as described by King et al. and Naguib et al (King et al., 2022; Naguib et al., 2023). The library construction and sequencing were undertaken by Novogene (Cambridge, United Kingdom). Geneious Prime work package 2024.0.7 (Biomatters, Auckland, New Zealand) was used for alignment and annotation of the obtained reads. Additionally, Kraken2 (Wood et al., 2019) was used to analyse the outcomes reads, followed by data visualization using web visualization tool Krona (Ondov et al., 2011). Phylogenetic tree of the HA gene segments of HPAI H5N1 viruses from Laos were constructed after the selection of the best-fitted model, by employing maximum likelihood methodology based on Akaike criterion using IQ-tree online tool https://iqtree.github.io. Branch support was assessed using 1000 ultrafast bootstrap replicates, and the resulting tree was visualized using FigTree v1.4.4.

### 2.4 Statistical analysis

Statistical analysis was performed using Stata 14.2 (StataCorp, Texas, USA). Results were summarized descriptively. Univariable comparisons between categorical variables were done using Pearson χ^2^ test, Fisher’s exact test (depending on if assumptions for χ^2^ test were met), and one-way analysis of variance (ANOVA) with Bonferroni corrections. Binomial exact calculations were used for 95% confidence intervals (95% CI). When multiple variables were associated with the occurrence of the virus, multivariable logistic regression was done using the command logit, with manual backward elimination. A p-value less than 0.05 was considered significant.

## 3 Results

### 3.1 Prevalence of AIV and CoVs in LWM

This study comprised 266 samples from 20 live and wet markets in the provinces of Vientiane capital and Vientiane province in Laos. Eleven markets were characterized as small (1–14 vendors), four as medium (15–100 vendors), and five as large (>100 vendors). 2.6% (n=7) of the collected samples were environmental swabs, 9.0% (n=24) were air samples, 17.7% (n=47) were tracheal swabs from slaughtered poultry, of which 95.7% (n=45) were from chickens, and 4.3% (n=2) were from ducks. The remaining 70.7% (n=188) were tracheal swabs from live birds, of which 67.6% (n=127) were chickens, 30.3% (n=57) were ducks, and 2.1% (n=4) were guinea fowls. All 266 samples were analysed for AIV and CoV by RT-qPCR. During sampling no obvious symptoms of disease (e.g. respiratory or nervous signs, cyanosis, ruffled feather) were observed. All interviewed vendors sold either live (45%, n=24) or dead (40%, n=21) poultry, or both (15%, n=8). Vendors were asked if the birds they sold had ever been vaccinated, and 20.8% said that they had been vaccinated, 34% stated that they had not been vaccinated, and the rest did not know. The type of vaccination was not asked.

**Figure 2:**
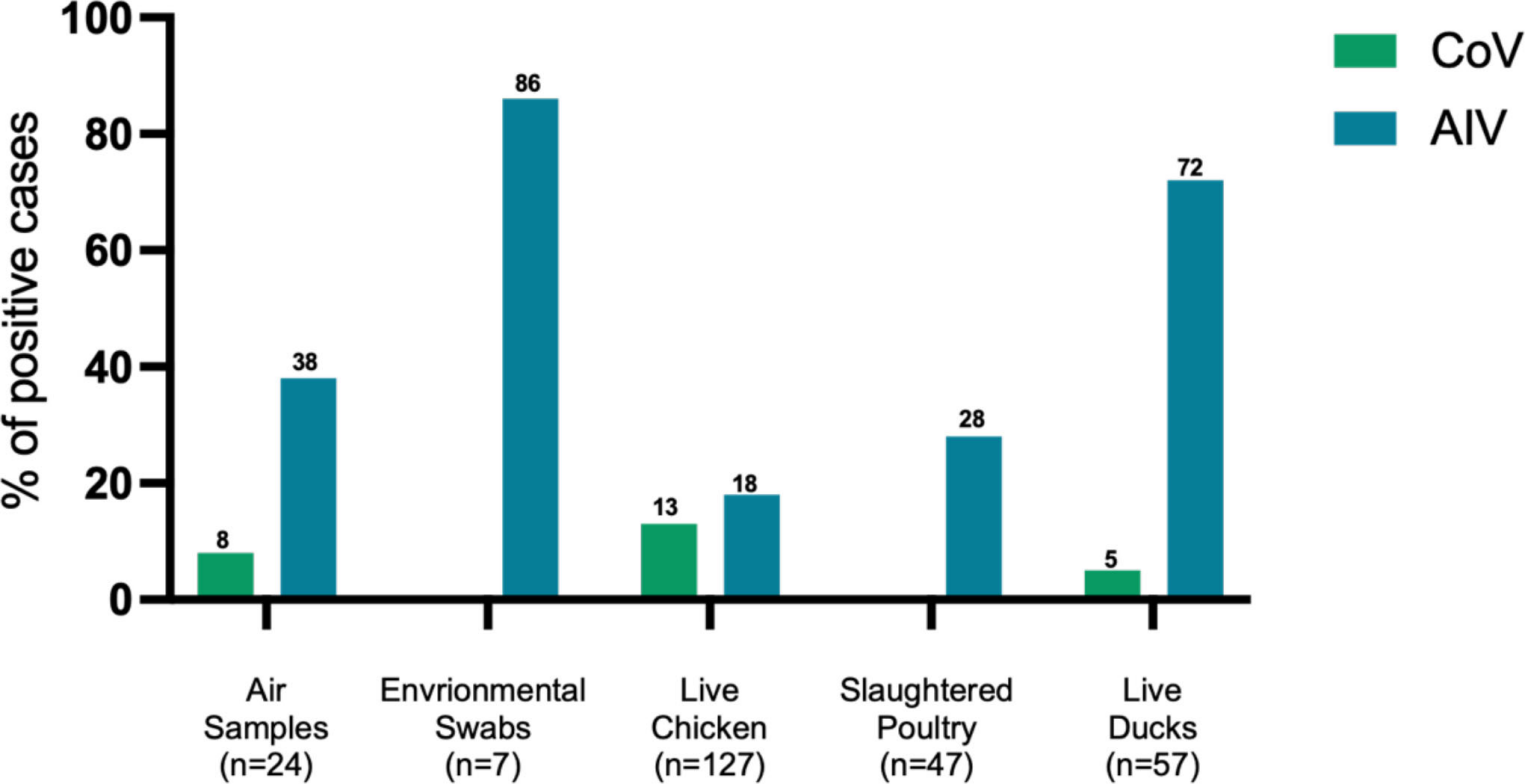
Overview of the sample types and their positive rates for CoV and AIV. The Y axis indicates the number percentage of positive AIV (blue) and CoV (green) cases in collected samples. The total number of collected samples for each category is indicated in brackets.

The prevalence of AIV was determined in all sample types. Of 266 samples, 35.3% (n=94, 95% CI 29.5-41.4%) were positive for AIV. Divided by sample type, 85.7% (n=6) of environmental swabs, 37.5% (n=9) of air samples, 27.6% (n=13) of swabs from slaughtered poultry, 71.9% (n=41) of swabs from live ducks, and 18.1% (n=23) of swabs from live chickens were positive for AIV (**Figure 2**; **Table 1**). Additionally, two of the four guinea fowl samples were positive. In total 35% (n=66) of all live birds were positive. Although only six environmental swabs were tested, they were significantly more likely to be positive than live- and slaughtered poultry samples (p = 0.030). Ducks were more likely to yield positive samples than chickens (p < 0.0001). Cycle threshold (Ct) values for AIV-positive samples varied across sample types, with the majority exceeding a Ct value of 30, indicating generally low viral loads. However, a subset of samples, particularly from live ducks, exhibited Ct values below 30, suggesting higher viral RNA concentrations in these cases (**Supplementary Figure 1**). In contrast, Ct values of CoV-positive samples were consistently above 30, with no indication of high viral loads. These findings reflect overall lower CoV burden compared to AIV in the sampled population and support the observed differences in prevalence.

When correlating market type (small, medium, large) with the total percentage of positive samples for AIV, it can be seen that in small-sized markets the proportion of positives was significantly *(p* = 0.010) higher (44%) than in medium-sized markets (24%). Small-sized markets also had a higher prevalence of AIV compared to large-sized markets; however, no significance was found. A multivariable analysis showed that market type, and types of poultry tested were both significant predictors (**Supplementary Table 2**).

All samples were screened for CoVs using an RT-qPCR. Across all 266 samples, 8.3% (n=22) tested positive for CoV, as seen in **Table 1**. CoV was found in 8.3% (n=2) of air samples, 13.4% (n=17) of swabs from live chickens, and in 5.3% (n=3) of swabs from live ducks. Three of all total positive live birds were positive for both AIV and CoV, and all three of these samples were from ducks. No significant differences between the sample types were found. This is probably due to the lower number of positives. All air samples were additionally subjected to a SARS-CoV-2 specific PCR, none of the samples were positive.

When correlating the market type to the total percentage of positive samples of CoV, medium-sized markets had a significantly higher prevalence (15.3%) compared to large-sized markets (2.0%) (p = 0.030). The prevalence was also higher in medium markets than in small-sized ones, but this difference was not significant, and small-size markets were also not significantly different from large markets.

### 3.2 Subtyping and genetic characterization of avian influenza virus

To determine the subtype of the hemagglutinin gene in a selected number of samples, a subset of 31 samples was chosen based on the virus load and sample quality typically samples with Ct value being <30 in the initial pan-avian influenza virus PCR. Those samples were run on three different RT-qPCRs, each specific for one subtype: IAV/H5, IAV/H7, IAV/H9. Of the 31 samples, 15 (48%) tested positive for H5, and 16 (52%) were not positive for any of the tested subtypes and are considered a different subtype, these were not investigated further.

**Figure 3:**
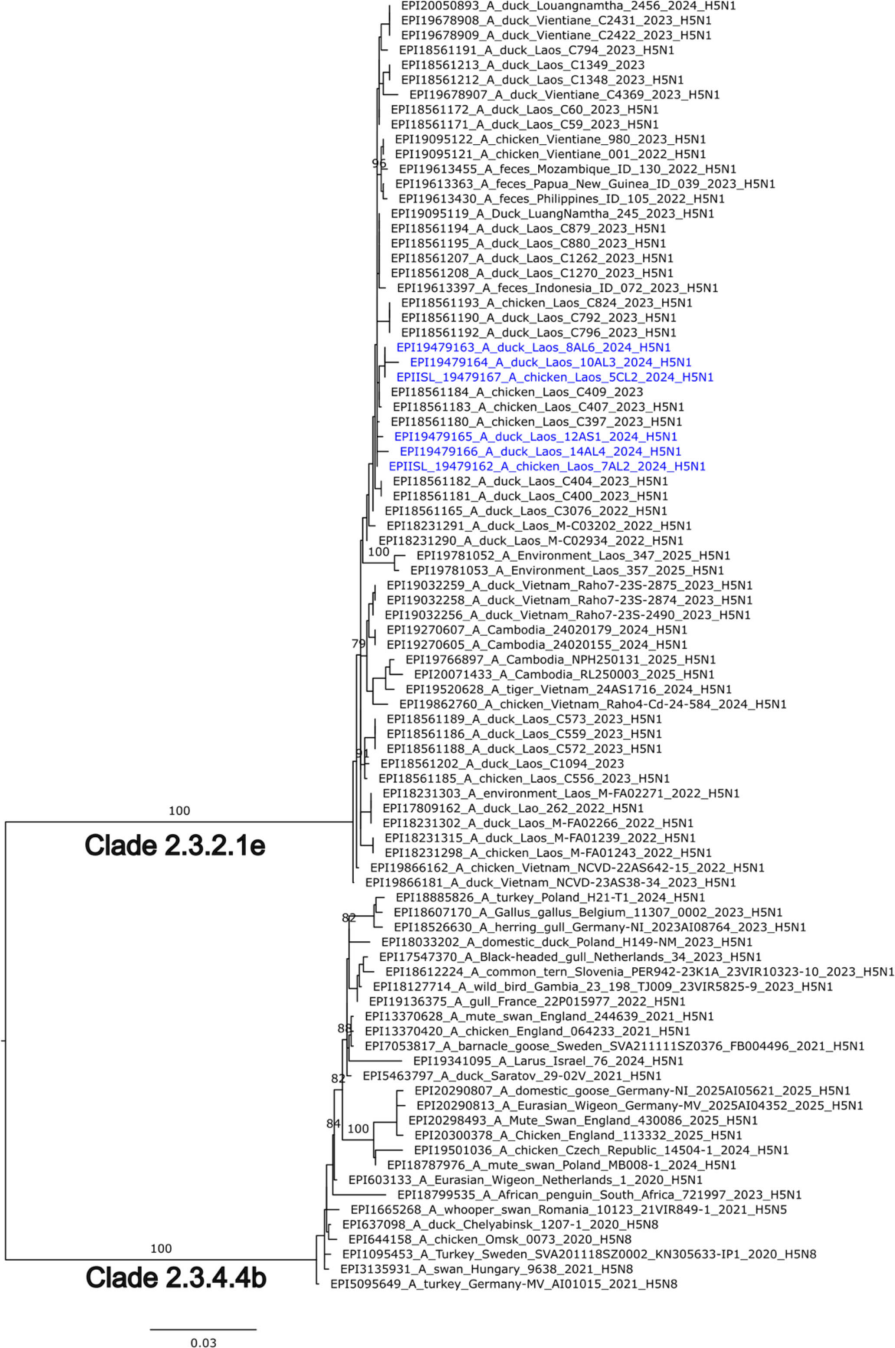
Phylogenetic tree of the HA gene segments of HPAI H5N1 viruses from Loas. Viruses detected in this study are shown in blue. Representative reference strains from clade 2.3.2.1e and 2.3.4.4b are shown in black. The tree was generated, after the selection of the best-fitted model, by employing maximum likelihood methodology based on Akaike criterion using IQ-tree online tool https://iqtree.github.io. Branch labels indicate bootstrap support values calculated from 1000 replicates and are shown in black.

Data retrieved from genome sequencing revealed a total of 9–12 million reads per sample from six samples with virus reads below 0.04%. The six samples showed partial sequence of influenza A virus genes with 99% similarity to the recent HPAIV H5N1 of clade 2.3.2.1e reported in Laos in 2023 (A/chicken/Laos/C409/2023, GISAID accession number EPI2807639). Phylogenetic analyses were found consistent with the genetic similarity and showed that the HPAI H5N1 viruses from Laos clustered with viruses from clade 2.3.2.1e (**Figure 3**). Sequences of the HA (n=6) and NA (n=6) generated in this study were made publicly available at the GISAID platform under isolate ID EPI_ISL_19479162 to 67 and accession numbers EPI3600848–53 for the HA and EPI4679876-8, EPI4679884, EPI4679888–9 for the NA.

### 3.3 Knowledge and practices of market actors

A total of 51 vendors and 32 customers were interviewed (**Supplementary Table 3**). Women had a higher representation than men across vendors (Women = 73%, Men = 27%), whilst for customers, it was more equally distributed (Women = 47%, Men = 53%). For both customers and vendors, the most frequent age group was 30-59 (percentage in this age group: customers = 50%, vendors = 67%), followed by 15-29 (customers = 34%, vendors = 20%), and ≥60 (customers = 3%, vendors = 10%). When asked about education, 19% had no formal education, 59% had gone to school and 22% to university. 92% of all people who did not go to school, and 57% of all who went to university were female. Of all vendors, 28% had no formal education.

47% of vendors (n=16) stated that they had seen birds with “flu-like symptoms” before. A follow-up question revealed that the majority (61%, n=19/31) said they would deal with a diseased animal by killing and burying it. Two respondents reported eating a diseased animal. Around a quarter (n=8) say they would do nothing. When asked about how vendors deal with deceased animals, the answer was even clearer, with 100% (n=31) stating they dispose of it themselves. Only two of these vendors said that they additionally informed the authorities.

All interviewees were questioned regarding their knowledge of zoonotic diseases and their concern about disease risks at the wet markets. As seen in **Supplementary Table 4**, more than half knew that animals could transmit diseases to humans, had heard about avian influenza/bird flu, and were in general concerned about diseases at the markets. Furthermore, the educational level was compared to the answers. It can be seen that the higher the formal education, the more people answered that they do know about zoonotic diseases and AIV, and were also more concerned about the disease risks at markets. When further questioned about which diseases the concern is mainly about, 53% (n=20/38) answered with COVID-19, and 16% (n=6) were concerned about influenza/bird flu. The rest of the answers were mainly malaria or diarrhoea. The interviewees were also asked about the safety of buying live animals at markets, around two-thirds (65%, n=54/83) answered that they view it as safe, and only 4% (n=3) see it as a risk. When asked if they did anything to protect themselves in the markets, 61.3% said they washed hands, and 78.7% stated that they wore face masks.

When asked about the effect of the COVID-19 pandemic on their markets, 70% (n=27/53) of the vendors answered that their market was highly affected. Considering that 81% (n=43) of the vendors stated that the income they earn at the markets contributes all (n=23) or almost all (n=20) of the total household income, it is clear that the markets are of utmost importance to the livelihood of people and the local economy. This was further underlined by the fact that 91% (n=73/80) of all interviewees agreed with the statement that many people would lose their jobs and income if markets were closed.

## 4 Discussion

Live and wet markets (LWMs) are traditional markets frequently selling live animals, particularly poultry (Kogan et al., 2019). The continuous interaction between humans and live animals at these markets increases the risk of zoonotic transmission (CDC, 2019; Piret and Boivin, 2021). Both AIV and CoV, have caused global outbreaks in the past, some originating from LWMs (CDC, 2019; Naguib et al., 2021; Piret and Boivin, 2021). Therefore, it is essential to continuously monitor viruses in LWMs. This study aimed to estimate the prevalence of AIV and CoV at LWMs in Laos, specifically in the capital region. Additionally, it assessed the knowledge and practices towards infectious diseases among market vendors and shoppers.

More than 35% of all live birds sampled tested positive for AIV. This prevalence was higher than the rates reported in neighbouring countries, being more than double the prevalence found in Myanmar and almost five times more than that observed in Vietnam (6.9%) (Chu et al., 2017; Borkenhagen et al., 2023). The prevalence of AIV in air samples was found at a higher rate compared to that found in other neighbouring countries e.g. Myanmar (Borkenhagen et al., 2023). Due to the limited number of environmental swabs, a comparison to other studies is not possible. Hence, future studies are recommended to incorporate environmental swabs alongside other specimens to evaluate their effectiveness for market sampling. This approach offers several advantages: it is cost-effective, as it requires no specialized equipment; it minimizes interactions with live animals, addressing ethical and biosafety concerns; and it streamlines the overall sampling process.

AIV samples with high virus load were subsequently subjected to HA subtyping, revealing that about half (48%) of the subtyped samples are of the H5 type. The remaining 16 samples that tested negative in our study were specifically negative for H9 and H7 subtypes. However, these samples may harbour other HA subtypes that were not included in our analysis. Previous studies in the South-East Asian region have shown individual cases of other AIV subtypes than H5/H7/H9, this includes for example H3, H6, and H15 (El-Shesheny et al., 2018; Dao et al., 2024). Based on the sequencing results, the H5 positive samples were found H5N1 clade 2.3.2.1e, which has previously caused significant avian outbreaks in SEA and human infections and fatalities, most recently in Cambodia in 2023 (Park et al., 2020; European Centre for Disease Prevention and Control, 2023). Interestingly, even while half of the samples did not have one of the haemagglutinin (HA) subtypes tested for, the results are showing that at least two different subtypes were circulating at the same time. All of this might suggest that Laos is an understudied hotspot for AIV spreading in LWMs. One limitation of this study is that whole-genome sequencing was not conducted for all samples as well as NA identification, which may have constrained our ability to analyse potential transmission events or reassortment in greater detail. Hence, future studies incorporating a broader range of sample types and whole-genome and phylogenetic analyses could provide a more complete understanding of the epidemiology and evolutionary dynamics of these viruses. Ducks and chickens may have different roles in CoV maintenance and transmission, and this study found an overall positive rate of 10.8% for CoVs in live birds, with 5% in ducks, and 13% in chickens, which is also similar to a study in live poultry in Laos (Pauly et al., 2019). This aligns with expectations, since avian coronaviruses, such as IBV, are highly prevalent amongst chickens worldwide (Bhuiyan et al., 2019; Al-Khalaifah et al., 2022). Although the protocol used in this study allows for the identification of CoV-positive samples, it also carries the potential of detecting non-avian CoVs, including human-origin CoVs. Additionally, the detection of CoV in air samples (8%) is noteworthy and suggests a potential role of airborne transmission in live bird markets. A study conducted in Bangladesh reported a 17.5% prevalence of IBV, an avian CoV, in chickens across the country, which is similar to the findings in this study in Laos (Bhuiyan et al., 2019). While our study does not directly assess transmission pathways, the presence of CoV in some air samples raises concerns about possible exposure risks.

Interestingly, the prevalence of AIV in live ducks was found to be four times higher (72%) than in live chickens (18%). This highlights the need for targeted surveillance and control measures in markets with a high proportion of ducks to prevent potential spillover events from wild to domestic birds. Another result of this study was the prevalence of AIV in slaughtered poultry. 28% of all slaughtered poultry tested positive for AIV, whilst none were positive for CoV. It has been previously reported that human infections can occur from slaughtered poultry (Li et al., 2022). This challenges the view that formal markets, which do not sell live animals, are safer for consumers, and highlights the need for more intensive research on this topic. In light of these observations, implementing appropriate biosecurity measures is important to reduce potential exposure risks. In particular, minimizing exposure risks via fomites, such as poultry products, can be achieved through proper handling and strict hygiene practices during processing and preparation.

When correlating the total percentage of positive samples to the market types, we found that AIV was more prevalent in small markets than in medium or large ones. One possible explanation is that smaller markets tend to have more birds crowded into each cage, increasing the risk of transmission (Lin et al., 2021). Additionally, larger markets appeared to implement better hygienic practices, such as more frequent cage washing, which could reduce virus spread (Lin et al., 2021). These observations were made during sampling, but future studies should systematically investigate the factors that differentiate markets. Additionally, small markets had a higher proportion of ducks, which are more likely to carry AIV. Furthermore, medium-sized markets showed a significantly higher prevalence of CoV compared to small markets, which potentially could be attributed to higher bird density, more contamination, and potentially poorer biosecurity routines, however, these factors were not assessed. One of the greatest risk sources of aerosolization is de-feathering machines frequently used across markets in Southeast Asia (Zhou et al., 2016). While this study did not quantify the use of such machines, it was observed that they were widely used at LWMs in Laos, particularly in smaller markets. Since smaller markets showed higher AIV risk, future research should explore if de-feathering machines contribute to this, and if they could be replaced by other methods.

Poultry vaccination against avian influenza viruses is not routinely practiced in Laos, and only 20% of the vendors said that their birds had been vaccinated (Boltz et al., 2006). There can be several challenges with vaccination of backyard and small-scale poultry in low-and middle-income countries, including cold chains and the fact that poultry vaccines often are sold in vials meant for many birds (Alders et al., 2007; Lindahl et al., 2019). A study by Islam et al. (2023) demonstrated that common and inexpensive preventive measures, like daily cleaning and weakly disinfection, can drastically reduce the risk of AIV transmission (Islam et al., 2023). Many disinfectants, including chlorine-based disinfectants, quaternary ammonium compounds, and phenolics, have effect on AIV, but thorough mechanic cleaning is important to reduce the pathogen load and allow the disinfectants to work (Khalil et al., 2023). Environmental persistence of viruses may depend on different factors, such as temperature and humidity, where AIV can remain infectious for several days in poultry faeces (Kurmi et al., 2013; Pawar et al., 2018) (up to 50 days based on the pH) or feathers (up to 160 days based on the temperature) (Yamamoto et al., 2010) compared to coronaviruses, which last for few days (e.g. four days on plastic surfaces) (Ashokkumar et al., 2023). While basic hygiene practices were observed during this study, a comprehensive, systematic biosafety plan at the market level would be necessary to reduce the risks of transmission. Implementing such measures could prevent market closures, an action also shown to reduce transmission risk, and thus avoid the associated loss of income for vendors, while still effectively mitigating the risk of AIV spread (Islam et al., 2023).

None of the air samples analysed tested positive for SARS-CoV-2. Detection of SARS-CoV-2 in air samples would have been of importance, indicating the presence of SARS-CoV-2 RNA in the air at the markets, although this does not necessarily reflect the presence of infectious virus. However, as of August 2022, 69% of Laos’ population was fully vaccinated, a rate that exceeded the global average for lower middle-income countries (59%) but is on the lower end compared to other countries in SEA (Mathieu et al., 2020). Nonetheless, Laos has achieved the World Health Organization’s target of a 70% vaccination rate (World Health Organization, 2021). During the sampling period, the 7-day rolling average of daily new confirmed COVID-19 cases per million people in Laos ranged from 0.47 to 0.02. However, data on the number of tests conducted during this time is not available (Mathieu et al., 2024), but this likely indicates a very low circulation of the virus and explain why all samples were negative.

The most common age of vendors (30-59) in this study aligns with a nationwide study reporting an average age of 39.9 years, with similar education levels across regions, indicating no significant variation in age or education among vendors in Laos (Philavong et al., 2020). The vast majority of vendors reported that they handle a diseased animal by either culling and burying it or by consuming it. Additionally, all vendors indicated that they dispose of deceased animals themselves, with only a small number informing the authorities. These answers align with previous research highlighting this issue as a persistent problem in Southeast Asia, particularly in Laos (Philavong et al., 2020). Moreover, mass mortality of birds during poultry rearing has become normalized among many farmers due to the increasing prevalence of AIV and Newcastle disease over the past two decades. However, without the vendors or farmers informing the authorities, there is no possibility of establishing reliable disease surveillance or taking proactive measures in case of new emerging diseases (Annand et al., 2021). Unsurprisingly, higher formal education was associated with greater knowledge of zoonotic diseases. These findings indicate that enhancing public education on zoonotic diseases, particularly through early formal education, could significantly improve risk perception, and therefore reduce the likelihood of outbreaks by promoting safer practices. In the survey, many respondents said they would wash hands and wear masks, but most respondents were not wearing masks during the survey. This may indicate that the respondents knew that they should do this, and the response may be influenced by desirability bias.

Overall, the findings of our study reveal that live and wet markets may still be associated with potential risks to human health. Additionally, markets have a huge economic impact and people often rely on it as the sole source of income. Given their substantial cultural importance and economic impact, understanding the transmission risks of AIV and CoV infections can aid in preventing the future outbreaks. Strengthening hygiene protocols in live and wet markets is crucial to minimize the potential for virus transmission. Additionally, public awareness aimed at raising awareness of AIV risks, especially among bird handlers, could help reduce the likelihood of zoonotic spillover. Implementing preventive strategies e.g. vaccination could mitigate the risk of future zoonotic outbreaks and improve overall public health.

## Data Availability

Sequences of the HA (n=6) and NA (n=6) generated in this study were made publicly available at the GISAID platform under isolate ID EPI_ISL_19479162 to 67 and accession numbers EPI3600848–53 for the HA and EPI4679876-8, EPI4679884, EPI4679888–9 for the NA.

## Supplementary material

Supplementary Figure 1 Ct values of Avian Influenza Virus (AIV) and Coronavirus (CoV) detected in different sample types. Each point represents an individual positive qPCR result for either AIV (triangles) or CoV (circles) across all sample types.

Supplementary Table 1 Sequences of all nucleotide-based reagents.

Supplementary Table 2 Results of a multivariable model for avian influenza virus in markets in Laos, and prevalence AIV according to market and animal type.

Supplementary Table 3 Distribution of female and male shoppers and vendors across different age groups.

Supplementary Table 4 Results of the questionnaire on knowledge and concern about disease transmission from animals to humans among respondents with different levels of formal education.
