# Supplementary material for: Avian influenza and coronaviruses in live animal and wet markets in Laos: prevalence and public health considerations
Source: Front Cell Infect Microbiol. 2026 Apr 7;16:1786183. doi: 10.3389/fcimb.2026.1786183 (PMC13095770; doi:10.3389/fcimb.2026.1786183)
Supplement: Supplementary Figure 1 — Ct values of Avian Influenza Virus (AIV) and Coronavirus (CoV) detected in different sample types. Each point represents an individual positive qPCR result for either AIV (triangles) or CoV (circles) across all sample types. [file Supplementaryfile1.docx]

# Supplementary Material

# **Avian Influenza and Coronaviruses in Live Animal and Wet Markets in Laos: Prevalence and Public Health Considerations**

Supplementary figure 1. Ct values of Avian Influenza Virus (AIV) and Coronavirus (CoV) detected in different sample types. Each point represents an individual positive qPCR result for either AIV (triangles) or CoV (circles) across all sample types.


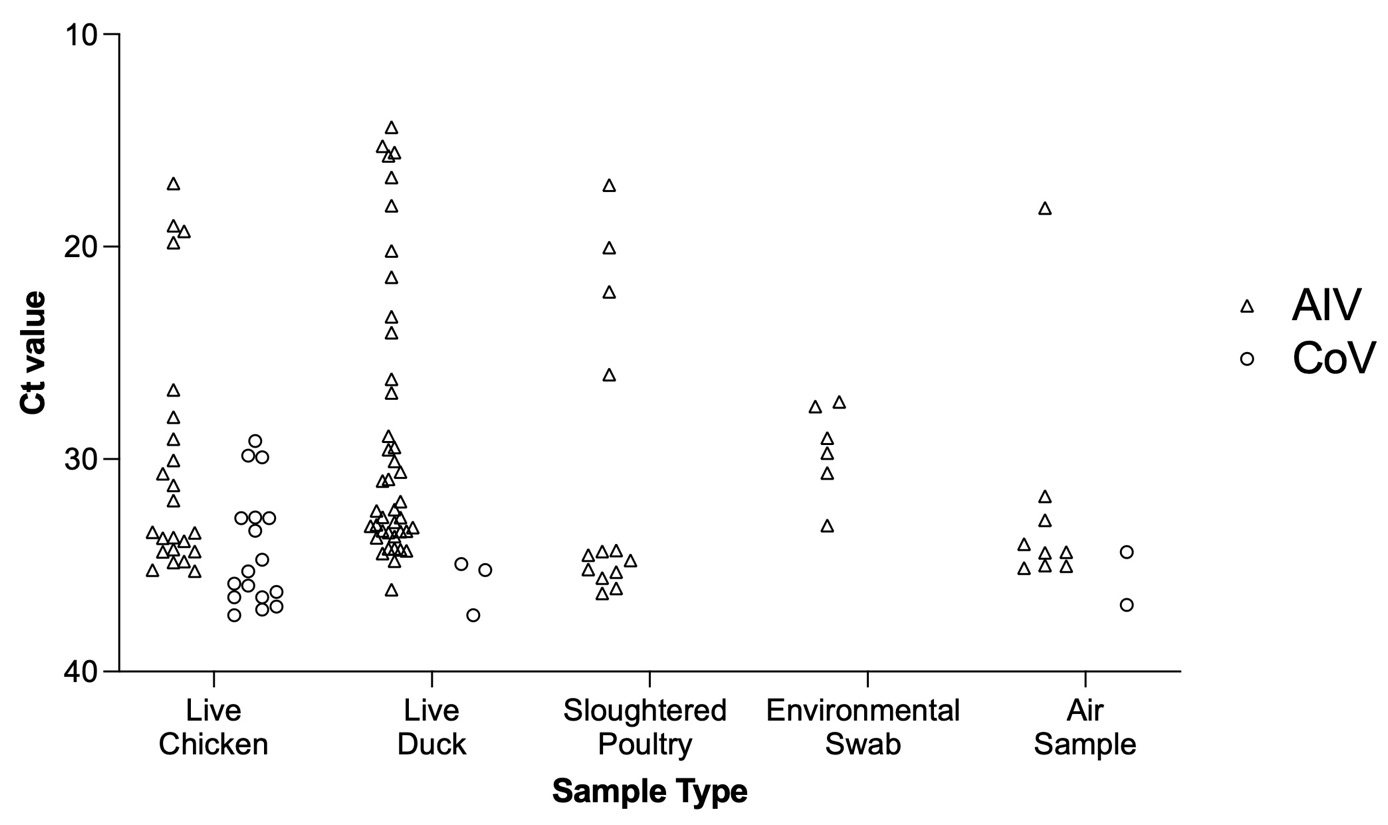


Supplementary Table 1: Sequences of all nucleotide-based reagents

| **Label** | **Sequence** | **Reference** |
| --- | --- | --- |
| *Avian Influenza Virus M RT-qPCR* | | (Spackman, 2020) |
| AIV forward | 5’ - AGATGAGTCTTCTAACCGAGGTCG - 3’ |  |
| AIV reverse | 5’ - TGCAAAAACATCTTCAAGTCTCTG - 3’ |  |
| AIV probe | 5’ - [FAM]TCAGGCCCCCTCAAAGCCGA[BHQ-1] - 3’ |  |
| *AIV whole genome amplification RT-PCR* | | (King et al., 2022) |
| WGS forward | 5’ - TCCCAGTCACGACGTCGTAGCGAAAGCAGG - 3’ |  |
| WGS reverse | 5’ - GGAAACAGCTATGACCATGAGTAGAAACAAGG - 3’ |  |
| *H5 specific RT-qPCR* | | (Hassan et al., 2022) |
| H5-HA1-F | 5’ - GATTYTAAARGATTGTAGYGTAGC - 3’ |  |
| H5-F2 | 5’ - GTTCCCTAGYAYTGGCAATCAT - 3’ |  |
| H5-HA-R1 | 5’ - CTCTCYACCATGTARGACCA - 3’ |  |
| H5-HA-R2 | 5’ - CTCTCYACTATGTARGACCA - 3’ |  |
| H5-R3 | 5’ - AATTCTARATGCAAATTCTGCAYTG - 3’ |  |
| H5-FAM3-RC | 5' - [6-FAM]CGCACATTGGRTTYCCRAGGAGCC[BHQ-1] - 3’ |  |
| H5-FAM2 | 5' - [6-FAM]CTGGTCTATYYTTRTGGATGTGCTCC[BHQ-1] - 3’ |  |
| *H7 specific RT-qPCR* | | (Hassan et al., 2022) |
| H7-F forward | 5’ - CAACTGAAACRGTRGARCG - 3’ |  |
| H7-R1 | 5’ - CAGGAGYCCACATTGACC - 3’ |  |
| H7-R2 | 5’ - CAGWAGYCCACATTGACC - 3’ |  |
| H7-R3 | 5’ - TTCTAGGAATTGGTCACATTG - 3’ |  |
| H7-FAM probe | 5’ - [FAM]CCCAGGATYTGCTCAARAGGRAAAA[BHQ-1] - 3’ |  |
| *H9 specific RT-qPCR* | | (Hassan et al., 2022) |
| H9 forward 1 | 5’ - CAATGGGGTTYGCTGCCT - 3’ |  |
| H9 forward 2 | 5’ - CAATGGGRKTTGCTGCCT - 3’ |  |
| H9 reverse | 5’ – TTATATACARATGTTGCAYCTG - 3’ |  |
| H9 probe | 5’ - [FAM]TTYTGGGCCATGTCIAATGGRTC[BHQ-1] - 3’ |  |
| *Coronavirus RT-qPCR* | | (Muradrasoli et al., 2009) |
| CoV forward | 5’ - TGATGATGSNGTTGTNTGYTAYAA - 3’ |  |
| CoV reverse | 5’ - GCATWGTRTGYTGNGARCARAATTC - 3’ |  |
| CoV probe_I | 5’ - [FAM]TTGTATTATCAGAATGGYGTSTTYATG[EDQ] - 3’ |  |
| CoV probe_III | 5’ - [FAM]TCTAARTGTTGGGTDGA[EDQ] - 3 |  |
| *SARS-CoV-2 RT-qPCR* | | (Corman et al., 2020) |
| SARS-CoV-2 forward | 5’ - ACAGGTACGTTAATAGTTAATAGCGT - 3’ |  |
| SARS-CoV-2 reverse | 5’ - ATATTGCAGCAGTACGCACACA - 3’ |  |
| SARS-CoV-2 probe | 5’ - [FAM]ACACTAGCCATCCTTACTGCGCTTCG[BBQ] - 3’ |  |

##

Supplementary Table 2: Results of a multivariable model for avian influenza virus in markets in Laos, and prevalence AIV according to market and animal type.

| Predictor | Odds ratio | 95% confidence interval | p-value | AIV prevalence |
| --- | --- | --- | --- | --- |
| Market type |  |  |  |  |
| Medium | Reference |  |  | 26.4 % |
| Large | 3.62 | 1.30-10.06 | 0.014 | 30.0 % |
| Small | 2.44 | 1.05-5.67 | 0.038 | 43.1 % |
| Animal type |  |  |  |  |
| Chicken | Reference |  |  | 18.1 % |
| Duck | 12.26 | 5.87-25.57 | <0.001 | 71.9 % |
| Guinea fowl | 9.00 | 1.08-74.64 | 0.042 | 50.0 % |
| Constant | 0.11 | 0.05-0.25 | <0.001 |  |
| *****Reference levels chosen to represent the lowest prevalence category | | | | |

Supplementary Table 3: Distribution of female and male shoppers and vendors across different age groups.

|  | Female | Male |
| --- | --- | --- |
| Shopper | 28.8% (15) | 54.8% (17) |
| 15–29 | 9.6% (5) | 19.4% (6) |
| 30–59 | 11.5% (6) | 32.3% (10) |
| ≥ 60 | 1.9% (1) | 0.0% (0) |
| No answer | 5.8% (3) | 3.2% (1) |
| Vendor | 71.2% (37) | 45.2% (14) |
| 15–29 | 15.4% (8) | 6.5% (2) |
| 30–59 | 46.2% (24) | 32.3% (10) |
| ≥ 60 | 9.6% (5) | 0.0% (0) |
| No answer | 0.0% (0) | 6.5% (2) |
| Total | 100% (52) | 100% (31) |

Supplementary Table 4: Results of the questionnaire on knowledge and concern about disease transmission from animals to humans among respondents with different levels of formal education.

| Formal Education | Do you know that animals can transmit diseases to humans? | | Do you know about avian influenza? | | Are you concerned about disease risk at the wet markets? | | |
| --- | --- | --- | --- | --- | --- | --- | --- |
|  | Yes % (#) | No  % (#) | Yes  % (#) | No  % (#) | Yes  % (#) | No  % (#) | Don’t know  % (#) |
| No formal Education | 50.0% (6) | 50.0% (6) | 54.5% (6) | 45.5% (5) | 58.3% (7) | 33.3% (4) | 8.3% (1) |
| School | 57.9% (22) | 42.1% (16) | 77.3% (17) | 22.3% (5) | 60.5% (23) | 39.5% (15) | 0.0% (0) |
| University | 78.6% (11) | 21.4% (3) | 80.0% (4) | 20.0% (1) | 71.4% (10) | 28.6% (4) | 0.0% (0) |
| No answer | 66.7% (14) | 33.3% (7) | 46.7% (7) | 53.3% (8) | 42.9% (9) | 52.4% (11) | 4.8% (1) |
| Total | 62.4% (53) | 37.6% (32) | 64.2% (34) | 35.8% (19) | 57.6% (49) | 40.0% (34) | 2.4% (2) |
